# Ultrasound-guided Axillary Vein Puncture for Cardiac Device Implantation: A Safe and Effective Approach

**DOI:** 10.19102/icrm.2023.14045

**Published:** 2023-04-15

**Authors:** Stefano Maffè, Paola Paffoni, Francesco Di Nardo, Luca Bergamasco, Eleonora Prenna, Emanuela Facchini, Giulia Careri, Nicolò Franchetti Pardo, Anna Maria Paino, Pierfranco Dellavesa

**Affiliations:** ^1^Division of Cardiology, SS Trinità Borgomanero Hospital, ASL NO, Novara, Italy; ^2^Medical Direction, SS Trinità Borgomanero Hospital, ASL NO, Novara, Italy

**Keywords:** Axillary vein, cardiac implantable electronic devices, ultrasound-guidance

## Abstract

Ultrasound-guided axillary vein access is an effective alternative to conventional subclavian and cephalic access for cardiac implantable electronic device implantation. The aim of this study was to compare the safety, efficacy, and radiation exposure data of the ultrasound-guided axillary approach with other conventional access techniques. The study population included 130 consecutive patients, stratified as 65 (64% male; median age, 79 years) in the study group and 65 (66% male; median age, 81 years) in the control group. We performed a retrospective not-randomized analysis by comparing ultrasound-guided axillary vein puncture with subclavian and cephalic approaches in order to test the effect on X-ray exposure, total procedure time, and complications. Significant differences were observed in terms of radiation exposure, including fluoroscopy time (median, 95 s [study group] vs. 193 s [control group]; *P* < .001), air kerma (median, 29 mGy [study group] vs. 55.7 mGy [control group]; *P* < .001), and dose–area product (median, 8219 mGy·cm^2^ [study group] vs. 16736 mGy·cm^2^ [control group]; *P* < .001). The median procedure time was 45 min in the study group but 50 min in the control group (*P* < .05). Complications occurred in 6 control group patients (1 urticaria contrast medium–related, 3 pneumothorax, 2 subclavian artery puncture) and 2 study group patients (2 axillary artery puncture). We conclude that the ultrasound-guided axillary venous approach is a fast, feasible, and safe technique for cardiac lead implantation. It allows a significant reduction in fluoroscopy time without prolonging the procedural time. This approach offers direct visualization of the vessel during the puncture, so it can be useful in patients who cannot receive contrast medium, those who require “difficult” thoracic approaches (emphysema, too much or too little fat tissue), or those on anticoagulant therapy.

## Introduction

Various venous access routes are used for permanent pacemaker (PM) or implantable cardioverter-defibrillator (ICD) implantation, including via the cephalic, axillary, and subclavian veins and internal and external jugular veins. A European Heart Rhythm Association survey showed that cephalic vein dissection and blind subclavian vein puncture are the preferred techniques for the implantation of cardiac implantable electronic device (CIED) leads in European centers.^1^ Cephalic vein dissection has the advantage of avoiding a central venous puncture and its complications, but it is highly dependent on the venous anatomy, the vessel caliber, and the operator’s surgical skills.^2^ Lead insertion failure rates range from 10%–70%, with failure attributed to the absence of the vein or its size being unable to accommodate multiple leads.^3^

The subclavian vein puncture technique, introduced by Littleford in 1979,^4^ is the most widely practiced, as it is relatively easy to learn, is quick to perform, and offers high success rates. However, it may cause potentially serious acute complications, including pneumothorax, hemothorax, and brachial plexus injury,^5–7^ or longer-term complications, such as lead fracture (due to or not due to subclavian crush) and/or insulation breaches, especially with ICD leads.^8^

Axillary vein puncture has emerged as a technique for the placement of pacing and defibrillation leads because the axillary vein is almost always large enough to accommodate multiple pacing leads, and it is completely extrathoracic, with a less angulated course.^9^ Axillary vein access using fluoroscopic landmarks is an effective approach for permanent PM and ICD implantation^10^ but is associated with a low but significant risk of arterial puncture, pneumothorax, or failed access, and this risk is also found with subclavian access.^11^ In contrast, recent results have shown that the use of ultrasound (US)-guided axillary access (USAA) is a safe and effective alternative to the conventional vein approach,^12^ enabling safe and effective puncture and requiring a shorter learning curve. We previously observed differences in procedural times and exposure to X-rays between USAA and other puncture approaches,^13^ but these differences could not be confirmed in a multivariate analysis that took into account potential confounders (such as body mass index [BMI] and body surface area, anticoagulant/antiplatelet treatment). The aim of the present study was to compare the safety, efficacy, and radiation exposure data of USAA with those of other conventional access routes.

## Methods

A retrospective analysis of all consecutive patients who had undergone either US-guided axillary vein puncture (study group) or any other approach (control group) for PM or ICD implantation between June 2020 and June 2021 was performed in order to test the effect of USAA on procedural time and radiation exposure. Patients with a history of previous CIED implantation and those undergoing cardiac resynchronization therapy were excluded. Only patients aged >18 years and capable of expressing their informed consent were considered eligible and entered the statistical analysis. All procedures were performed in the electrophysiology laboratory under standard sterile conditions by 3 experienced electrophysiologists trained in all venous sites of access. Neither fellows nor trainees acted as primary operators. Antibiotic prophylaxis (cephazolin or intravenous vancomycin) was administered 1 h before the procedure. In patients taking warfarin, the procedure was performed under a therapeutic international normalized ratio. Non-vitamin K oral anticoagulants were interrupted 24–48 h before the procedure, depending on the creatinine clearance, and resumed the day after the procedure. No patient received bridging anticoagulation therapy. The total procedural time was calculated from the time of the axillary vein puncture to the surgical suture of the pocket.

**Figure 1:**
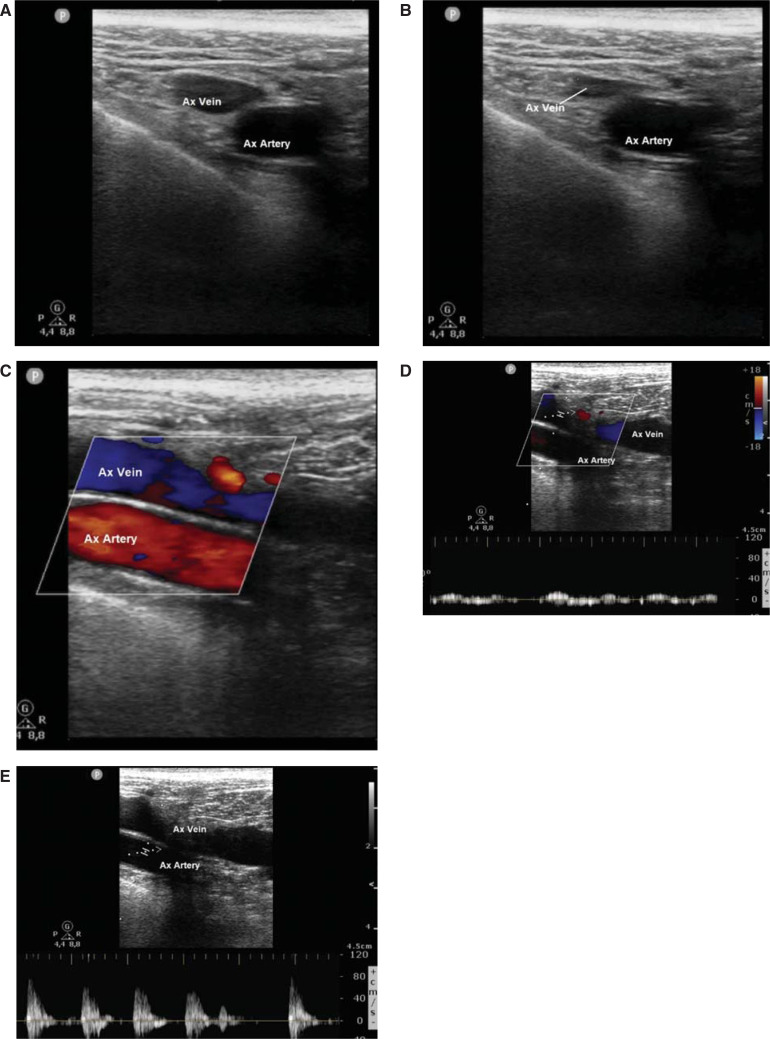
The axillary vein is easily distinguishable from the axillary artery owing to its compressibility **(A, B)**, more medial and superficial position **(C)**, and lack of pulsation **(D, E)**. *Abbreviation:* Ax, axillary.

US characteristics that make the axillary vein easily distinguishable from the axillary artery include its compressibility, lack of pulsation, and more medial and superficial positioning **(Figure 1)**. To perform the axillary approach, patients were placed in the supine position with their arms at their sides. The infraclavicular region was cleaned, and sterile surgical drapes were applied. A US machine (Philips CX50; Philips Healthcare, Eindhoven, the Netherlands) in vascular mode with a high-frequency linear transducer probe L12-3 (7 MHz) was used. To maintain sterility, the US probe was covered in a dedicated sterile plastic cover, and sterile gel was applied directly to the skin. Local anesthesia was administered with 1% lidocaine along the incision line and at the puncture site. The extrathoracic portion of the vein was prescanned in the long and short axes to determine the optimum puncture site. Care was taken to distinguish the artery from the vein: with gentle pressure, the vein could be easily compressed.

**Figure 2:**
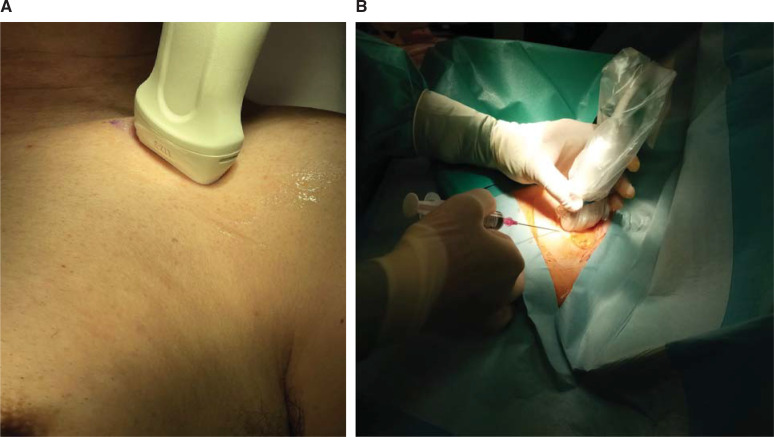
**(A)** Transducer, operator, and **(B)** needle configuration for ultrasound-guided axillary vein access in a draped representative patient. Cannulation was attempted with the ultrasound probe aligned perpendicular to the longitudinal axis of the axillary artery and vein.

**Figure 3:**
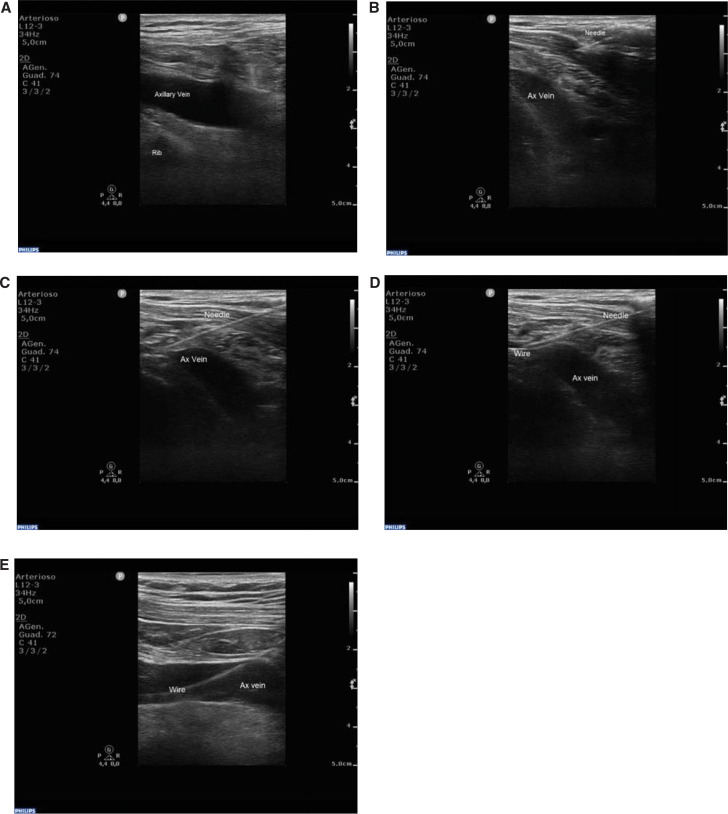
The longitudinal view of the axillary vein allows the operator to track the needle in the subcutis up to the anterior venous wall **(A–C)**, and to monitor insertion of the wire into the vessel **(D)**. Once the needle was seen to enter the vein, the syringe was removed and a 0.035′′ J-tipped guidewire was inserted into the vein and advanced with gentle pressure, without fluoroscopic guidance but under ultrasound guidance **(E)**. *Abbreviation:* Ax, axillary.

While holding the probe with the non-dominant hand, the operator performed the puncture with the dominant hand, using an 18-gauge needle. Cannulation was attempted in-plane with the longitudinal axis of the axillary vein, which is an approach that allows the operator to track the pathway of the needle in the subcutis up to the anterior venous wall and to monitor the insertion of the wire into the vessel **(Figure 2)**. A single- or double-puncture technique, retaining the guidewire, was used to implant a pair of leads. An 18-gauge, 7-cm-long bevel-tipped needle (Cook Medical, Bloomington, IN, USA) was inserted and advanced toward the vein; the operator kept their right hand below the US probe while watching for tissue movement on the US screen and maintaining negative pressure on the plunger. Once the needle was seen to enter the vein and blood flashed into the syringe, the syringe was removed and a 0.035′′ J-tipped guidewire was inserted into the vein and advanced with gentle pressure, without fluoroscopic guidance but under US guidance with a longitudinal view **(Figure 3)**.

Cross-sectional US images of the carotid artery and internal jugular vein were taken to confirm that the guidewire had not been inadvertently inserted into the internal jugular vein. The position of the guidewire in the superior vena cava was confirmed fluoroscopically. Once vascular access had been achieved, the wire was secured with vascular clamps outside the body. The skin was incised near the pectoral groove, and the device pocket was created. Surrounding tissues were dissected to allow the guidewire to be identified; the guidewire was then pulled to the pocket region. A peel-away sheath was used to insert the lead. An unsuccessful attempt was defined if the axillary vein was not cannulated after 10 min or in the event of venospasm or no-progression of the wire. The learning curve associated with USAA consisted of a self-learning period, was 15 patients for each operator, and was completed prior to the start of the study. In patients who failed the axillary approach, the 10 min consumed in the attempt was not considered in the calculation of the total procedural time so as to avoid inserting a bias into the evaluation of the procedural time in the control group. Other investigators consider larger caseloads of 25–30 patients for each operator.^12^

In the control group, or in the case of failure of axillary vein cannulation, a first rib subclavian approach or cephalic vein dissection was performed at the operator’s discretion. During the cephalic vein approach, after surgical exposure of the vein, direct lead insertion through a small venotomy was attempted. The intrathoracic subclavian vein was punctured with the help of conventional anatomic landmarks, as described by Littleford et al.^4^; if venous access could not be obtained, the operator could perform a contrast-guided venipuncture. The choice of approach in a given patient was operator-dependent.

The major complications considered in our study included death, cardiac arrest, cardiac perforation, cardiac valve injury, coronary venous dissection, hemothorax, pneumothorax, transient ischemic attack, stroke, myocardial infarction, pericardial tamponade, and arteriovenous fistula, while minor complications included drug reaction, conduction block, accidental artery punction, hematoma or lead dislodgement requiring reoperation, peripheral embolus, phlebitis, peripheral nerve injury, and device-related infection.

### Statistical analysis

A descriptive analysis of the sample was carried out using median and interquartile range (IQR) values for continuous variables and absolute and relative frequencies for qualitative variables. Non-parametric measures of statistical dispersion were chosen because of the non-normal distribution of the procedural times.

In order to test the effect of the type of approach (USAA vs. other approaches) to PM or ICD implantation on procedural time and exposure to X-rays, multivariate linear regressions were performed in which fluoroscopy time (s), air kerma (mGy), dose–area product (mGy·cm^2^), and procedural time (min) were the outcomes. Type of approach, age at implantation, sex, and factors with *P* < .25 in the univariate analysis were included in the multivariate linear regression, as suggested by Hosmer and Lemeshow.^14^ Other variables tested for possible confounding were BMI, body surface area, type of device implanted, and anticoagulant and/or antiplatelet treatment. Results were expressed in terms of crude and adjusted unstandardized coefficients with 95% confidence intervals (CIs). The analysis was performed using SPSS software version 13.0 for Windows (IBM Corporation, Armonk, NY, USA), and statistical significance was set at *P* ≤ .05.

## Results

Between June 2020 and June 2021, 130 consecutive patients were assessed for eligibility. We analyzed the data from all 130 patients aged 49–94 years. Sixty-five patients (50% of the sample) underwent US-guided axillary vein puncture. The other 65 underwent subclavian vein puncture, a cephalic vein approach, or a cephalic plus subclavian vein approach (control group). Five patients in the control group had previously undergone an unsuccessful attempt at US-guided axillary vein cannulation (65/70; 93% of successful attempts). Reasons for failure were vasospasm (n = 2) or difficulty of axillary vein visualization (n = 3).

No patients with a dual-chamber device underwent a hybrid approach, with 1 lead implanted through the axillary vein and another implanted through the cephalic or subclavian vein. The main demographic and clinical characteristics of the patients enrolled in this study are summarized in **Table 1**. The median puncture time in those who underwent USAA was 120 s (IQR, 142 s; minimum, 15 s; maximum, 660 s).

Following adjustment for possible confounders, USAA correlated with all of the outcomes studied. USAA required significantly less fluoroscopy time (median, 95 s; IQR, 100 s) than the other puncture types (median, 193 s; IQR, 167 s; adjusted *P* < .001; see **Table 2**). X-ray exposure parameters were significantly lower in the study group. The median air kerma was 29.0 mGy in the study group, as opposed to 55.7 mGy in the control group (adjusted *P* < .001, see **Table 3**), and the median dose–area product was 8,219 mGy·cm^2^ in the study group compared to 16,736 mGy·cm^2^ in the control group (adjusted *P* < .001, see **Table 4**). The procedural time was also significantly shorter in the study group (median, 45 min; IQR, 16 min) than that in the control group (median, 50 min; IQR, 19 min; adjusted *P* < .05; see **Table 5**).

Complications occurred in 6 control group patients (major complications were recorded in 3 patients with pneumothorax; minor complications were recorded in 1 patient with urticaria caused by contrast medium without a history of previous allergies and 2 patients with accidental subclavian artery puncture) and in 2 study group patients (minor complications were recorded in 2 patients with accidental axillary artery punctures, which were resolved by manual compression and hemostasis). Three of the 130 patients enrolled in this study (2.3% of the study population) suffered from contrast medium allergy. No contrast medium was used in any patient in the study group, which was instead injected in 10 (15.3%) patients in the control group.

Another variable associated with the study outcomes was the number of electrodes implanted. This was highly expected, as implanting more electrodes inevitably increases the procedural time and thus the exposure to X-rays. Results from the univariate and multivariate analyses (linear regression) confirmed that BMI is an independent variable significantly correlated with a shorter duration of the total procedural time (adjusted coefficient, −0.6; 95% CI, −1.2 to −0.1; *P* = .026).

## Discussion

The aim of this study was to compare the safety, efficacy, and radiation exposure data of the USAA approach with those of the traditional approaches (subclavian and cephalic) that use fluoroscopy. USAA proved to be safer than other techniques, as it required significantly less radiation exposure, without increasing the procedural time. This dose-saving occurs because the entire fluoroscopy phase required for subclavian puncture or the attempt to advance the leads along the cephalic vein is skipped.

In our study, we achieved a 93% success rate in axillary vein cannulation, with only 5 patients requiring alternative approaches. Recently, Ahmed et al. reported that US-guided CIED implantation was successful in the vast majority (93%) of patients, and rare cases of unsuccessful cannulation were associated with right-sided implantation and greater venous depth.^15^

In patients who underwent an axillary approach, we never needed to perform venography or to use contrast medium; this approach is therefore a valid option for allergic patients or those suffering from kidney disease. Furthermore, the use of venography is only partially helpful, as the vein trajectory is visualized before, and not during, the puncture. Moreover, venography does not provide any information on vessel depth. By contrast, USAA provides direct visualization of the vessel and the surrounding structures during the puncture, and the operator can follow the progress of the needle in the subcutaneous tissue, check the depth of the vein, and avoid accidental puncture of the artery, thus minimizing the number of unsuccessful attempts and complications. Moreover, the time required for lead placement and venous access is shorter than that for fluoroscopy and venography.^16^ USAA enables all the phases of venous puncture and cannulation to be monitored, even in patients with “difficult” thoraxes (obese, super-thin) or those on anticoagulant therapy, thereby lowering the risk of hematomas and arterial puncture (after an adequate learning curve). In this regard, in our study, we recorded 2 subclavian arterial punctures in the control group and 2 axillary arterial punctures in the study group (both in early USAA procedures, when the learning curve was probably not yet complete); the difference lies in the manual compressibility of the axillary site, with a lower risk of hematoma.

An interesting aspect of our study is the significant correlation during multivariate analysis between BMI and the reduction in total procedural time. The mean BMI was identical in both study groups (26.4 kg/m^2^ in the study group vs. 26 kg/m^2^ in the control group); it becomes an independent variable because, in patients with higher BMIs, the axillary approach is probably quicker to perform. In our opinion, the US-guided approach is advantageous in patients with a “difficult” thorax, in whom cannulating the vessel is faster, safer, and more precise than searching for and isolating a deep cephalic vein or performing a blind puncture with only anatomical landmarks of the subclavian vein.

The subclavian access is the most frequently used approach. However, it potentially exerts mechanical stress (subclavian crush syndrome) on the implanted leads, potentially resulting in mechanical lead failure or vein occlusion.^9,17^ Compelling evidence has implicated the infraclavicular musculotendinous complex in mechanical lead failure and fracture of subclavian catheters.^18^ Compared to using the subclavian vein, the properly accessed axillary vein offers a less angulated course, avoiding all these problems. Additionally, subclavian access portends the risk of inadvertently accessing the non-compressible subclavian artery (which occurred in 2 cases in our control group) and the potential for increased mechanical stress on the lead or indwelling catheter as a result of crossing the subclavius muscle and the clavipectoral fascia.

The current trend is to implant under antithrombotic therapy, because the perioperative bridging of anticoagulation is associated with a higher risk of thromboembolic events. A previous study reported greater use of pressure dressings with USAA,^16^ which may suggest a higher risk of bleeding relative to that associated with the cephalic approach. In our experience, however, we did not record any bruising or cases of excessive bleeding in either group. The cephalic approach allows direct exposure of the vessel, which can therefore be cannulated without blind punctures; however, the insertion and advancement of the leads is not always simple and often requires the aid of fluoroscopy, with non-negligible radiation exposure. The results of the first randomized clinical trial to compare self-learned USAA and cephalic vein dissection for PM and ICD lead insertion demonstrated significant superiority of USAA for cardiac lead implantation, even when performed by operators with no previous experience with the technique.^19^

Unlike in other studies, in which cannulation of the axillary vein was performed with the US probe aligned perpendicular to the axis of the vein,^13^ we performed the whole procedure with the US probe aligned longitudinally to the vein, in order to monitor the entire procedure; this included from the skin puncture and the passage of the needle through the subcutis to the puncture of the anterior wall of the vessel and to tracking the insertion of the guidewire and evaluating its correct progression in an antegrade direction, as explained in the figures.

Since the first successful insertion of a temporary transvenous endocardial lead through the brachial vein by Furman and Schwedel in 1959,^20^ many technical advances have been described, culminating in the widespread use of the procedure. The use of the axillary vein for CIED implantation was first suggested by Byrd.^21^ Since then, various techniques of axillary vein puncture have been proposed, either with contrast venography to help axillary vein localization or with the aid of fluoroscopic anatomical landmarks only (axillary “blind” puncture).^22^ Recently, a caudal view with the fluoroscopy camera moved to 35° caudal allows the operator to see the anterior outline of the lungs, and this approach should facilitate axillary venous access and reduce the risk of pneumothorax (note, 0 cases of pneumothorax were reported in a prior study with 229 implants).^23^ The axillary vein approach should reasonably be used as the first choice, as it has been proven to be safe and effective.^9^ Despite these benefits, however, use of the axillary vein remains uncommon, primarily because of the lack of proper training in the puncture technique and concerns regarding its supposedly long learning curve and secondarily owing to a shortage of evidence supporting US-guided axillary vein puncture in PM or ICD lead implantation. In short, a widespread “axillary culture” is lacking.

Recent studies have evaluated the safety and benefits of axillary vein puncture for ICD lead implantation using fluoroscopic landmarks, especially the body surface of the second rib. While this technique avoids the use of contrast venography and acute and long-term lead complications, it involves exposing the operator to radiation and its inherent risk of cancer and cataract onset.^13^ US guidance can improve the success rate of axillary vein cannulation because it is an easy-to-learn approach that reduces the risks of lead crush and pneumothorax. Moreover, it can be used in patients with pre-existing leads.^11^

The efficacy of USAA has been evaluated to date in 2 randomized clinical trials. In a relatively small (only 88 patients enrolled) prospective multicenter study, Tagliari et al. demonstrated that the axillary approach was superior in terms of success rate and procedural time compared to cephalic vein dissection, with a similar complication rate.^19^ In a larger study (174 patients), Liccardo et al. compared the efficacy of USAA to subclavian vein access. These authors concluded that success rates and complication rates were similar, and they proposed axillary access as a safe and effective alternative technique to the conventional subclavian vein access.^12^

Our experience is mainly focused on radiation exposure; to the best of our knowledge, this is one of the few studies on radiation exposure during CIED implantation using different approaches (axillary US-guided, subclavian, cephalic). Previous studies have shown that adopting a US-guided axillary venous access route involves less radiation exposure than using an axillary access route with fluoroscopic anatomical landmarks.^13^ Our study enrolled a larger sample of patients and is the first to take into account many potential confounders in a multivariate analysis, thus making the observed differences more convincing.

We firmly believe that axillary vein puncture performed under US guidance should always be the first choice for CIED implantation, as it improves the quality of patient care without increasing the procedural time.

### Study limitations

Our study has some limitations, the main one being that it was a single-center study involving only 3 operators and a relatively small patient sample; thus, our results may not be reproducible in centers with a different workflow. Second, the study was retrospective, and assignment to the study groups was not randomized. Furthermore, the operators themselves were the investigators, which might have resulted in significant bias. The fact that the choice of the approach was left to the judgment of the operator, based on their own experience and habits, without a well-specified selection criterion, represents a significant bias.

In this study, the existence of 3 pneumothorax cases among 65 patients is excessive and does not reflect the typical complication rate of the center; however, in this limited series, these were the data recorded. Cases of pneumothorax do not concern patients in whom the US-guided approach has failed.

Finally, a longer follow-up period would be needed in order to evaluate complications such as lead fracture, and different techniques could require different follow-up durations in order to assess the true incidence of adverse events.

We excluded patients undergoing cardiac resynchronization therapy device implantation from the study because our main objective was to demonstrate the reduction in fluoroscopic exposure that USAA entails. The implantation of a biventricular device already requires much longer fluoroscopy times, and this would have conditioned the statistical analysis of the results. In this sense, we are collecting a series of only this type of patient, which will be the subject population of future studies.

## Conclusions

US-guided axillary vein cannulation for CIED implantation is a feasible and safe alternative approach. Compared to the more widely used “blind” puncture technique using fluoroscopic anatomical landmarks, USAA offers direct visualization of the vessel during the puncture and a significant reduction in fluoroscopy times, without prolonging the procedural time. Thus, in conclusion, we believe that physicians should be sensitized toward the systematic use of US guidance for CIED implantation.

## Figures and Tables

**Table 1:** Main Demographic and Clinical Characteristics of Enrolled Patients

Characteristics	Control Group (n = 65)	Study Group (n = 65)
Age, years, median (IQR)	79 (13)	81 (12)
Sex, male, n (%)	42 (64.6)	43 (66.2)
BMI, kg/m^2^, median (IQR)	26.00 (4.70)	26.40 (4.80)
BSA, m^2^, median (IQR)	1.86 (0.27)	1.89 (0.26)
Implanted electrodes, n (%)		
* *1	26 (40.0)	34 (52.3)
* *2	39 (60.0)	31 (47.7)
Creatinine, mg/dL, median (IQR)	1.00 (0.42)	1.00 (0.53)
Device type, n (%)		
* *PM	56 (86.2)	56 (86.2)
* *ICD	9 (13.8)	9 (13.8)
Puncture, n (%)		
* *SVP	35 (53.8)	—
* *CVA	25 (38.5)	—
CPS	5 (7.7)	—
Contrast, n (%)	10 (15.3)	0 (0.0)
Allergies, n (%)	2 (3.1)	2 (3.1)
Anticoagulant treatment, n (%)	26 (40.0)	27 (41.5)
Antiplatelet treatment, n (%)	23 (35.4)	21 (32.3)
DAPT	0 (0.0)	4 (6.2)
Complications, n (%)	6 (9.2)	2 (3.0)
Overall procedural time, min, median (IQR)	50 (19)	45 (16)
Fluoroscopy time, s, median (IQR)	193 (167)	95 (100)
Air kerma, mGy, median (IQR)	55.7 (48.4)	29.0 (29.3)
Dose–area product, mGy·cm^2^, median (IQR)	16,736 (15,176)	8219 (8160)

*Abbreviations:* BMI, body mass index; BSA, body surface area; CPS, cephalic plus subclavian veins; CVA, cephalic vein approach; DAPT, double antiplatelet treatment; ICD, implantable cardioverter-defibrillator; IQR, interquartile range; PM, pacemaker; SVP, subclavian vein puncture; US, ultrasound. Comparison is control versus US-guided axillary vein puncture.

**Table 2:** Correlation Between Fluoroscopy Time (s) and the Variables Analyzed

	Crude Coefficient (Crude *P*)	Adjusted Coefficient	95% CI, Lower/Upper Bounds	Adjusted *P*
Approach (US-guided axillary vein puncture)	−114 (<.001)	−103	−144/−63	<.001
Age	0 (.972)	1	−2/2	.840
Sex (male)	8 (.724)	10	−34/54	.660
BMI	−6 (.059)	−3	−9/2	.222
BSA	−14 (.821)			
Device type (ICD)^a^	−18 (.586)			
Anticoagulant treatment	−30 (.195)	−22	−63/19	.294
APT	12 (.622)			
Number of electrodes	65 (.004)	52	11/93	.013

*Abbreviations:* APT, antiplatelet treatment; BMI, body mass index; BSA, body surface area; CI, confidence interval; ICD, implantable cardioverter-defibrillator; US, ultrasound. Results are from the univariate and multivariate analyses (linear regression). The type of approach to gain venous access, age on treatment, sex, and factors with a *P* < .25 on univariate analysis entered the multivariate linear regression. ^a^Reference: pacemaker.

**Table 3:** Correlation Between Air Kerma (mGy) and the Variables Analyzed

	Crude Coefficient (Crude *P*)	Adjusted Coefficient	95% CI, Lower/Upper Bounds	Adjusted *P*
Approach (US-guided axillary vein puncture)	−29.8 (<.001)	−28.5	−41.4/−15.7	<.001
Age	0.0 (.981)	0.3	−0.4/1.0	.419
Sex (male)	6.8 (.369)	5.4	−9.5/20.2	.474
BMI	−0.7 (.482)			
BSA	32.6 (.096)	35.4	−3.6/74.4	.075
Device type (ICD)^a^	−3.6 (.730)			
Anticoagulant treatment	−6.0 (.416)			
APT	−5.6 (.466)			
Number of electrodes	23.8 (.001)	21.3	8.2/34.3	.002

*Abbreviations:* APT, antiplatelet treatment; BMI, body mass index; BSA, body surface area; CI, confidence interval; ICD, implantable cardioverter-defibrillator; US, ultrasound. Results are from the univariate and multivariate analyses (linear regression). The type of approach to gain venous access, age on treatment, sex, and factors with a *P* < .25 on univariate analysis entered the multivariate linear regression. ^a^Reference: pacemaker.

**Table 4:** Correlation Between Dose–Area Product (mGy·cm^2^) and the Variables Analyzed

	Crude Coefficient (Crude *P*)	Adjusted Coefficient	95% CI, Lower/Upper Bounds	Adjusted *P*
Approach (US-guided axillary vein puncture)	−9430 (<.001)	−9068	−13,158/−4977	<.001
Age	2 (.987)	91	−133/316	.423
Sex (male)	2188 (.360)	1838	−2877/6553	.442
BMI	−161 (.591)			
BSA	9381 (.130)	10,124	−2248/22,496	.108
Device type (ICD)^a^	−1137 (.730)			
Anticoagulant treatment	−2202 (.341)			
APT	−1143 (.635)			
Number of electrodes	7061 (.002)	6286	2136/10,435	.003

*Abbreviations:* APT, antiplatelet treatment; BMI, body mass index; BSA, body surface area; CI, confidence interval; ICD, implantable cardioverter-defibrillator; US, ultrasound. Results are from the univariate and multivariate analyses (linear regression). The type of approach to gain venous access, age on treatment, sex, and factors with a *P* < .25 on univariate analysis entered the multivariate linear regression. ^a^Reference: pacemaker.

**Table 5:** Correlation Between Procedure Time (min) and the Variables Analyzed

	Crude Coefficient (Crude *P*)	Adjusted Coefficient	95% CI, Lower/Upper Bounds	Adjusted *P*
Approach (US-guided axillary vein puncture)	−7.8 (.001)	−5.6	−9.6/−1.5	.007
Age	−0.2 (.134)	−0.2	−0.4/0.1	.089
Sex (male)	0.1 (.989)	0.2	−4.2/4.6	.925
BMI	−0.6 (.045)	−0.6	−1.2/−0.1	.026
BSA	−3.0 (.630)			
Device type (ICD)^a^	0.4 (.902)			
Anticoagulant treatment	−3.7 (.116)	−2.6	−6.7/1.5	.217
APT	1.7 (.480)			
Number of electrodes	11.0 (<.001)	9.9	5.8/14.0	<.001

*Abbreviations:* APT, antiplatelet treatment; BMI, body mass index; BSA, body surface area; CI, confidence interval; ICD, implantable cardioverter-defibrillator; US, ultrasound. Results are from the univariate and multivariate analyses (linear regression). The type of approach to gain venous access, age on treatment, sex, and factors with a *P* < .25 on univariate analysis entered the multivariate linear regression. ^a^Reference: pacemaker.
